# Rheumatic complications of checkpoint inhibitors: Lessons from autoimmunity

**DOI:** 10.1111/imr.13242

**Published:** 2023-07-12

**Authors:** Gary Reynolds

**Affiliations:** ^1^ Institute of Cellular Medicine Newcastle University Newcastle upon Tyne UK; ^2^ Center for Immunology and Inflammatory Diseases Massachusetts General Hospital Boston Massachusetts USA

**Keywords:** arthritis, autoimmunity, checkpoint inhibitors, CTLA4, immunotherapy, PD1, rheumatology

## Abstract

Immune checkpoint inhibitors are now an established treatment in the management of a range of cancers. Their success means that their use is likely to increase in future in terms of the numbers of patients treated, the indications and the range of immune checkpoints targeted. They function by counteracting immune evasion by the tumor but, as a consequence, can breach self‐tolerance at other sites leading to a range of immune‐related adverse events. Included among these complications are a range of rheumatologic complications, including inflammatory arthritis and keratoconjunctivitis sicca. These superficially resemble immune‐mediated rheumatic diseases (IMRDs) such as rheumatoid arthritis and Sjogren's disease but preliminary studies suggest they are clinically and immunologically distinct entities. However, there appear to be common processes that predispose to the development of both that may inform preventative interventions and predictive tools. Both groups of conditions highlight the centrality of immune checkpoints in controlling tolerance and how it can be restored. Here we will discuss some of these commonalities and differences between rheumatic irAEs and IMRDs.

## 1 INTRODUCTION

Immune checkpoint inhibitors (ICIs) are a therapeutic class of treatments that reinvigorate immune responses to tumor neoantigens. Their use has resulted in improved outcomes in a range of cancers and they are capable of inducing durable remission in some cases. The first in class therapy was ipilimumab which targets the CTLA4 axis and was approved for the treatment of melanoma in 2011.^1^ Following this initial success the range of indications has expanded with a further seven treatments targeting PD1/PDL1 receiving FDA approval.^2^ As of January 2023, there were 1610 active or recruiting clinical trials in checkpoint inhibitors (ClinicalTrials.gov) with 156 of these at Phase III/IV. Given this it is likely that the range of immune checkpoints targeted and the number of patients receiving checkpoint inhibitor therapies will increase significantly over the next decade. However, a significant barrier to their broader application is the development of immune‐mediated adverse events (irAEs). These comprise a heterogeneous group of treatment‐associated effects mediated by autoreactive immune responses that are distinct from the side‐effects of conventional treatments. They can affect nearly any organ and are considered an aberrant off‐target response by an excessively activated immune system.^3^ They are very common, occurring in nearly 80% of patients treated with combination therapy^4^ and many patients develop more than one toxicity.^5^

**FIGURE 1:**
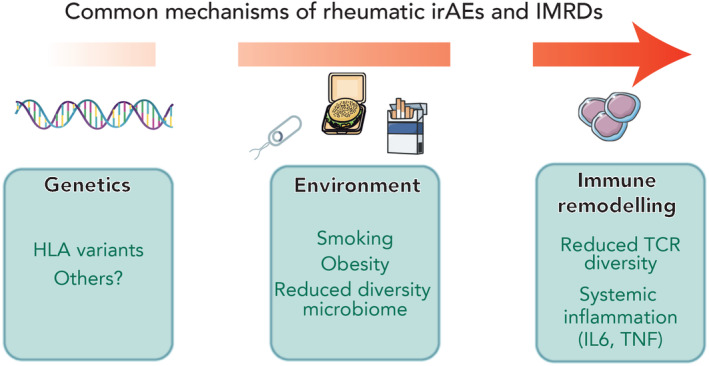
Common mechanisms underlying rheumatic irAEs and IMRDs. Multiple factors that are known to contribute to the development of common IMRDs have been shown to predispose to the development of rheumatic irAEs, including genetic and environmental factors. Similarly there are shared alterations in peripheral immunity that herald the development of both.

Among these complications are a range of rheumatic toxicities that includes inflammatory arthritis, keratoconjunctivitis sicca, xerostomia, a polymyalgia‐like illness, and myositis. In contrast to severe complications such as myocarditis, rheumatic irAEs are rarely fatal. However, they are disabling, they can limit treatment efficacy, they can become chronic and they are likely to become more common. Furthermore, while rates of rheumatic irAEs with current therapies targeting the CTLA4 and PD1/PDL1 pathway are low at around 5%, both primary human immunodeficiencies and animal models suggest modulating different immune checkpoints may produce different patterns of autoimmune complications, potentially with higher rates and severity of rheumatic diseases. Rheumatic irAEs share superficial similarities with common immune‐mediated rheumatic diseases (IMRDs) such as rheumatoid arthritis (RA), psoriatic arthritis (PsA), Sjogren's syndrome (SS), and polymyalgia rheumatica (PMR), for example in clinical presentation, that make it tempting to group them together. Indeed, in the absence of robust characterization and evidence, applying lessons from IMRDs to rheumatic irAEs has been an effective approach to guide management. However emerging evidence highlights that they are clinically and immunologically distinct processes.^6^ Despite this, they highlight the importance of immune checkpoints in self‐tolerance and there are intriguing commonalities in the factors that lead to the development of both (Figure 1). Here we will discuss these, reviewing the parallels and differences between them that may help us understand how they develop.

## 2 PARALLELS IN THE DEVELOPMENT OF IMRDS AND RHEUMATIC IRAES

In contrast to rheumatic irAEs, the clinical presentation of IMRDs usually represents the culmination of decades of subclinical altered immunity in genetically predisposed individuals.^7^, ^8^ Many IMRDs have a preclinical phase characterized by detectable self‐reactive responses, for example circulating auto‐antibodies against citrullinated peptides in RA (anti‐citrullinated peptide antibodies, ACPAs) and against nuclear components in Sjogren's and SLE (anti‐nuclear antibodies, ANA), that are present many years before the onset of disease. In seronegative conditions such as psoriatic arthritis it is harder to define this preclinical phase of autoreactivity but, for example, there is a median period of 8 years between the onset of psoriasis and the development of psoriatic arthritis suggesting a similarly prolonged period of subclinical autoreactivity.^9^ The development of rheumatic irAEs is more abrupt, with a median time of onset across all irAEs from initiation of 63 days.^10^ However, this delay, and the fact that not everyone develops an irAE, demonstrates that ICI treatment is a necessary but not sufficient condition for the development of these complications. A better understanding of the additional risk factors that result in complications is important for assessing risk and tailoring therapy in future.

### 2.1 Genetics

Genetic factors contribute significantly to the development of IMRDs with heritability estimates of around 40%–60% for RA, SLE, and Sjogren's syndrome.^11^, ^12^, ^13^ Genome wide association studies (GWAS) have helped to identify many of the variants that convey protection or risk of developing these conditions. Of the non‐HLA variants detected these generally fall in distal regulatory regions and individually have small effect sizes. The complexity of the genetic basis of IMRDs makes it challenging to identify individuals at risk of developing disease to initiate preventative strategies such as lifestyle changes or immunomodulatory therapy. Polygenic risk scores (PRS) attempt to overcome this by summarizing the effect of all variants to provide an overall estimate of the genetic contribution to an individual's disease risk. These are complex and require large datasets, e.g., a recent RA PRS included 276,020.^14^ Despite this it is hoped that the same approach might be taken to predict individuals at risk of developing complications from ICI therapy. Like IMRDs, there is almost certainly a genetic predisposition, but the challenge of developing analogous PRSs may be even more daunting than for IMRDs. For example, conceivably the genetic risk variants that predispose to ipilimumab‐induced colitis are quite different to those that predispose to nivolumab‐induced arthritis. There may be hope that, much like there are common risk variants across autoimmune conditions such as *CTLA4*, *TNF*, and *PTPN22*,^15^ there are shared variants that predict ICI toxicity that can form the basis of a predictive score. Supporting this notion, a SNP in the *IL7* gene was associated with the presence of any form or grade of ICI toxicity in a study of 1751 patients,^16^ although this gene does not appear as a hit for IMRDs across GWAS. Similarly, early studies suggest that, much like IMRDs, different HLA alleles appear to predispose to different organ‐specific toxicities (reviewed in Ref. 17). In IMRDs it is theorized that HLA variants possess different binding affinities for self‐antigens; notably for example shared epitope alleles of RA having greater affinity for citrullinated peptides. The association of specific HLA variants with irAEs could similarly imply targeting of common autoantigens in individual rheumatic irAEs. As more detailed characterization of the genetic basis of rheumatic irAEs is developed, it will be interesting to see how it overlaps with that of IMRDs, if at all. Similarly, given the correlation between the development of irAEs and ICI efficacy, it will be important to determine the correlation between variants that predispose to toxicity and those that predict ICI efficacy.

### 2.2 Environment

The contribution of the environment to the development of IMRDs is complex and multifactorial. It includes variables such as diet, obesity, smoking, and infection. A commonality among some of these factors is that they can promote inflammation and neoantigen formation. In RA, mucosal inflammation induced by smoking in the lungs or periodontitis in the oral cavity upregulates the enzyme PAD which leads to the formation of citrullinated peptides. In SLE, UV light induces cellular apoptosis and inflammation leading to defective clearance and the generation of antibody responses to nuclear components such as double stranded DNA. Cocaine use results in nasal mucosal inflammation and a clinical picture that resembles vasculitis, including the development of antineutrophil cytoplasmic antibodies.

In contrast to rheumatic irAEs, which develop subacutely, these factors are generally presumed to influence IMRD development over long periods. However, there are some common risk factors, for example, both smoking and obesity increases the risk of irAE development.^18^, ^19^ A more immediate source of inflammation and neoantigen formation that may drive breach of tolerance may be the tumor microenvironment itself. Tumors with low levels of inflammation, as defined by the level of T‐cell infiltration, are described as “cold” and have poorer responses to ICIs. Mutations result in altered peptides that generate neoantigens and enhance the inflammatory response. Neoantigen generation as quantified by mutational burden of the primary tumor has been posited as a predictor of efficacy of ICI response,^20^ although evidence is mixed depending on definitions used.^21^ Conventional cytotoxic chemotherapy can enhance ICI responses, presumably as it can induce cell death and neoantigen formation within otherwise cold tumors.^22^ Given this, it might be hypothesized that tumor mutational burden would be associated with higher rates of rheumatic irAEs, as they contain the requisite environment of inflammation and neoantigen formation for their development of IMRDs. Meta‐analysis of anti‐PD1 responses potentially supports this with a correlation between rates of irAE and median number of somatic mutations per megabase in the tumor type.^23^

Microbiome is a key environmental factor influencing IMRD development, ICI efficacy and irAE development. It is potentially more amenable to preventative intervention than other risk factors. Promisingly fecal microbiota transplant has demonstrated efficacy in both improving efficacy of ICI therapy and in the treatment of ICI‐induced colitis.^24^ However, the host‐microbiome interactions responsible for this efficacy are unclear and across studies multiple taxa and species have been implicated in the development of response and toxicity with ICIs which makes tailoring therapy difficult. Generally, overall diversity of species appears to be important in determining outcomes, with high diversity of gut microbiota associated with a response to ICI treatment and antibiotic therapy with adverse outcomes. The same effect is seen in IMRD development with reduced gut microbiome diversity associated with the development of RA,^25^ SLE,^26^ psoriatic arthritis,^27^ and different forms of vasculitis.^28^ As for ICI response and toxicity, although individual species have been associated with different types and stages of IMRDs, there is minimal consistent overlap between them.^29^ Interestingly, different organ involvement of irAEs is associated with different patterns of gut microbiota, with multiple Streptococcus and Lactobacillus species specifically associated with the development of ICI‐arthritis rather than other toxicities.^30^ Although these associations are preliminary, species from the same genera have been associated with RA development.^31^

### 2.3 Aging

The risk of autoimmunity generally increases with age for many conditions, including RA. Aging itself is associated with a complex of immune alterations including raised inflammatory cytokines and declines in the diversity and increases in clonality of naive CD4 and CD8 T cells, a process termed “inflammaging”.^32^, ^33^ It might be posited that this would predispose to irAE development, and in murine models irAEs were commoner in older mice.^34^ Despite this, preliminary studies do not suggest a heightened risk in older people.^35^ Pediatric patients (<21 years old) treated with ipilimumab for solid organ tumors developed irAEs at a similar rate to adults.^36^ This may depend on the ICI and toxicity itself though, as in another study arthritis was notably commoner in older people.^37^

## 3 IMMUNE PHENOTYPING OF RHEUMATIC IRAES

### 3.1 T cells

To date there have been limited analyses of the immune profile of rheumatic irAEs. What is known so far suggests the mechanisms responsible for irAEs are different from those responsible for IMRDs, supporting the notion from epidemiological and clinical data that these are distinct conditions. In contrast there appear to be surprising similarities between irAE toxicities across organs. In irAE‐arthritis there is an expansion of *PDCD1* ^hi^ *CXCL13* ^+^ *CD8A* ^+^ T cells. These T cells are activated (expressing *IFNG* and *HAVCR2*), proliferative, and are more clonal.^38^, ^39^ Phenotypically similar activated *PDCD1* ^hi^ *CXCL13* ^+^ *CD8A* ^+^ T cells have been found to be enriched in irAE‐colitis,^40^ again expressing high *IFNG* and *HAVCR2* but, in contrast to those in irArthritis, also coexpressing Th17 cytokines *IL17A* and *IL26*. The same population is enriched in checkpoint inhibitor pneumonitis, again overexpressing IL17A and *IFNG* but also here expressing *CSF2*.^41^ Notably this conserved transcriptional signature of CD8 T cells is seen across tumors in response to checkpoint inhibitor therapy and is predicted to mark clonal responses to tumor neo‐antigens, with a similar phenotype identified within tumors and sites of metastasis.^42^ Alongside recognized prognostic indicators like mutational burden, CD8‐derived CXCL13 within tumors is an independent predictor of checkpoint inhibitor sensitivity.^43^ To date a meta‐analysis of cell states across irAEs or with their IMRD equivalents has not been made but it will be interesting to determine the degree of conservation between and across them.

In contrast there are parallels between the changes that take place in the T‐cell repertoire between rheumatic irAEs and IMRDs prior to clinical presentation. The mechanism of anti‐PD1 treatment is to invigorate existing T‐cell clones, and this induces a detectable oligoclonal expansion in the periphery with an overall reduction in diversity.^44^ However, findings vary between studies and measures of diversity are highly donor dependent so it is difficult to convert this observation into a tractable biomarker. In IMRDs the same pattern is a feature of pre‐ or early IMRDs with a reduction in diversity associated with oligoclonal expansions a feature of Sjogren's,^45^ PMR/GCA,^46^ and early RA.^47^ This likely represents affinity maturation and epitope spreading of the autoantibody responses leading to responses with greater specificity for targeted self‐antigens and at the same time antibodies recognizing a broader range of self‐antigens.^48^, ^49^

### 3.2 B cells

Despite the prominence of the naive B‐cell homing cytokine CXCL13 in irAE expression data, rheumatic irAEs are notable for the lack of B‐cell infiltrates. Many IMRDs are associated with autoantibodies that not only indicate the diagnosis but also help to stratify conditions by treatment responsiveness, organ involvement and prognosis. Histologically target organs of IMRDs, including RA synovium and ANCA‐vasculitis affected lungs, contain tertiary lymphoid structures that facilitate responses against local antigens.^50^, ^51^ By contrast rheumatic irAEs such as arthritis have limited B cell infiltration.^38^, ^39^ Labial salivary gland biopsies from irAE‐induced exocrine gland dysfunction superficially share a similar pattern of lymphocytic inflammation to Sjogren's, but they are notable for the reduced or absent level of CD20^+^ B cells.^52^ Plasma cells are present in muscle in inclusion body myositis and polymyositis, with evidence of affinity maturation with IgM to IgG isotype class switching, clonal expansion and somatic mutation within muscle.^53^ In contrast, in irAE myositis, there is a near absence of B cells and a predominance of CD8^+^ T cells.^54^ Notably, although B‐cell infiltration in irAEs is limited, in many primary tumors B cells and plasma cells are not only present but also positively correlate with response to treatment.^55^

### 3.3 Cytokine profiles

The development of targeted biologic therapies for the management of IMRDs has helped to reveal the cytokine networks underlying different conditions. In inflammatory arthritis for example, whereas IL6 blockade is effective in RA, it is ineffective in psoriatic arthritis. Similarly whereas anti‐IL17/23 treatment is effective in psoriatic arthritis, it is not effective in RA. Anti‐TNF therapy is effective in both psoriatic arthritis and RA but, in predisposed individuals, can induce the development of SLE. This highlights the importance of developing a robust taxonomy of inflammatory arthritis and other IMRDs using criteria such as serology, clinical presentation and associated clinical features. Currently most patients with rheumatic irAEs are treated with corticosteroids, as these are fast acting and broadly effective in inflammatory conditions. For some conditions, such as PMR, vasculitis and myositis, corticosteroids remain the mainstay of therapy as for their irAE mimics. However, corticosteroids have significant adverse side‐effects and, with the advent of targeted biologic therapies, long‐term therapy with them is avoided in IMRDs where possible. Second‐line treatment for many rheumatic irAEs is conventional DMARDs such as methotrexate and sulfasalazine. Methotrexate works through multiple mechanisms including inhibition of NF‐kB and increasing T‐cell sensitivity to apoptosis,^56^ and it is broadly effective in a range of IMRDs (and other forms of autoimmunity) so its efficacy in irAE arthritis does distinguish it. However, as there is a high risk of irAE‐arthritis persistence after cessation of treatment (around 49% at 6 months),^57^ there is likely to be greater use of more targeted therapies in future. To date multiple agents have shown efficacy, including anti‐TNF,^58^ anti‐IL6R (tocilizumab),^59^ and tofacitnib (JAK inhibitor),^60^ but numbers are small. There is evidence to suggest the IL17/23 axis is active in irAE‐arthritis with enrichment of Th17 cells, but there has been a reticence to target this pathway because of a concern that it can negatively affect anti‐tumor immunity.^61^ A potential outcome from the increased use of targeted therapies may be to highlight underlying heterogeneity underlying shared clinical presentations of rheumatic irAEs. For example anti‐PD1/PDL1 monotherapy commonly causes a small joint arthritis whereas in combination with anti‐CTLA4 a large joint mono/oligoarthritis is more common.^62^ Patients on combination CTLA4/PD1 therapy have higher levels of CD4^+^IL17^+^ T cells in both peripheral blood and synovial fluid than those on monotherapy, potentially indicating differentially active cytokine networks.^39^ Conceivably, as with IMRDs, these different presentations are driven by different cytokine networks with different responses to treatment.

## 4 CTLA4—A CENTRAL PATHWAY IN RHEUMATIC DISEASE AND CANCER

CTLA4 was the first immune checkpoint to be targeted for cancer immunotherapy. *CTLA4* is a significant GWAS loci in many autoimmune conditions including RA, Grave's disease, Type I diabetes mellitus, vitiligo, and alopecia areata.^63^ In CTLA4‐deficient mice, alongside multiorgan inflammation particularly of the heart and lungs, mice occasionally develop arthritis and vasculitis.^64^ Interestingly, as with rheumatic irAEs, the immune infiltrate in CTLA4 deficiency is predominantly T cells with relatively few B cells. In human CTLA4 deficiency, inflammatory arthritis occurs in around 14% of individuals (though notably other autoimmune complications are more common).^65^ As with irAE‐arthritis, while psoriatic arthritis and RA can occur in these patients, generally the inflammatory arthritis that develops is a clinically distinct entity.^66^ Abatacept, a CTLA4 fusion protein, was approved for use in rheumatoid arthritis in 2008 (5 years before the approval of ipilimumab^67^) and, following this, has subsequently demonstrated efficacy in many diverse IMRDs including psoriatic arthritis, juvenile idiopathic arthritis (JIA), myositis,^68^ giant cell arteritis (GCA)^69^ and ANCA vasculitis.^70^ Overall these data suggest that CTLA4 plays a central role in maintaining tolerance and, consistent with this, rates of irAEs are higher for the CTLA4‐targeting therapy ipilimumab than for other checkpoint inhibitors that target PD1/PD‐L1.

CTLA4 is upregulated upon activation by CD4^+^ and CD8^+^ T cells and constitutively expressed by Tregs. Its ligands, CD80 and CD86, which are the targets of abatacept, are more broadly expressed by antigen presenting cells, B cells, monocytes/macrophages, and tumor cells. This means that it is an oversimplification to state that abatacept has the opposite therapeutic effect of ipilimumab. Ipilimumab is thought to function by lifting the block on co‐stimulation of naive T cells, although this is debated.^71^ In support of this Ipilimumab does not alter existing antiviral and anti‐tumor responses, but instead promotes new anti‐melanoma responses and a broadening of the TCR repertoire.^44^, ^72^ In contrast, modulation of naive responses is less likely to be the mechanism of action of abatacept. It is as effective as other biologic agents such as anti‐TNFs in established IMRDs, long after initial priming has taken place.^73^ In collagen‐induced arthritis abatacept is still effective at ameliorating inflammation in the absence of CD4^+^ T cells in established disease.^74^

The function of CTLA4 in other cell types is less well understood than its action at the stage of initial naive T‐cell priming in secondary lymphoid organs. This mechanism makes it an attractive tool to modulate nascent immune responses through targeting CTLA4 in early or preclinical IMRDs such as RA and Sjogren's. Clinical trials of abatacept in very early disease have taken place with the outcome of preventing or delaying the development of disease or inducing remission.^75^, ^76^, ^77^ To date this approach has been disappointing; while there is an increasing rates of patients maintaining drug‐free remission, an effect that appears to be due to true immune modulation rather than simply anti‐inflammatory effects as it easily outlasts the effect of abatacept treatment (half‐life 14 days), the effects are small (14% vs. 8%), with the vast majority continuing to develop chronic disease. However, given the potential to prevent long‐term, disabling disease, there is ongoing enthusiasm in exploring whether better targeting of immune checkpoints, patient selection or the stage of disease could transform the trajectory of autoimmunity.

In this model of ipilimumab function, T cells specific for tumor neoantigens are present but due to the suppressive function of CTLA4 in secondary lymphoid organs fail to generate appropriate anti‐tumor responses. Given this, there would be understandable anxiety that abatacept would suppress normal tumor surveillance. Reassuringly longitudinal data collected across multiple national registries of biologic rheumatic drugs in real‐world cohorts do not highlight a risk signal for cancer with abatacept treatment.^78^, ^79^, ^80^ Abatacept is avoided with live vaccines but patients with RA on abatacept are able to mount reasonable vaccine responses^81^ and have a lower risk of infection than with other biologics.^82^

## 5 TARGETING DIFFERENT IMMUNE CHECKPOINTS: NEW FORMS OF RHEUMATIC IRAES?

Currently only the CTLA4 and PD1/PDL1 immune checkpoints are therapeutically targeted for the treatment of cancer. However, there are more than 30 different molecules that are considered immune checkpoints. These vary in distribution by cell type, activation status, and organ and their action can be activating or inhibitory. This diversity allows fine tuning of the immune response and, in a system in which around 4% of T cells are self‐reactive in the absence of regulatory T cells,^83^ vital to maintain self‐tolerance. Following the success of CTLA4 and PD1/PDL1 therapies, there has unsurprisingly been interest in targeting other immune checkpoints including BTLA, VISTA, TIM‐3, and CD47 as well as co‐stimulatory molecules such as CD137, OX40, and GITR^84^ with the hope that targeting other immune checkpoints may result in lower rates of toxicity or greater efficacy in a given cancer. The accumulated outcome data from anti‐CTLA4 and anti‐PD1/PDL1 trials have highlighted that targeting different immune checkpoints results in different rates of treatment efficacy and irAEs, but also that the patterns and clinical picture of the irAEs that develop differ. To date, in comparison to other forms of irAE, rheumatic irAEs are comparatively unusual, and generally seronegative with highest rates of inflammatory arthritis, polymyalgia and exocrine gland dysfunction. Presentations of irAEs that resemble potentially organ‐ and life‐threatening conditions like lupus or vasculitis are thankfully rare. As clinical trials progress our understanding of how targeting these different immune checkpoints influences the range of clinical presentations that can develop. However at this stage, with the recognition of the limitation of preclinical models to predicting irAEs, there are some indications of the pattern of rheumatic irAEs that could develop with these novel therapies.

### 5.1 VISTA

VISTA is an inhibitory checkpoint molecule expressed by lymphocytes, but also more broadly and at higher levels on myeloid cells. VISTA deficiency results in spontaneous development of cutaneous and systemic lupus‐like disease accompanied by ANA and anti‐double‐stranded DNA antibodies in non‐predisposed BALB/c mice.^85^ In contrast, although lupus can be exacerbated when CTLA4 is disrupted or induced in susceptible strains, it does not spontaneously occur and while glomerulonephritis and arthritis develop in PD‐1 knockout mice, this is not associated with the development of anti‐dsDNA antibodies.^86^ Interestingly, as well as immune cells VISTA is expressed at high levels of keratinocytes where it regulates IFN‐I production. Cutaneous UV exposure is an important trigger for SLE and it has been proposed that inhibiting VISTA may predispose to exacerbations or de novo development of SLE.^87^ Currently anti‐VISTA therapies for cancer are at preclinical or Phase I stages of testing.^88^, ^89^

### 5.2 TIM3

TIM3 is an inhibitory molecule that is broadly expressed across immune cells. It has multiple ligands including the alarmin HMGB1. In the presence of cell lysis, for example in the cancer microenvironment, immunogenic nucleic acids are released. TIM3 interacts with HMGB1 to prevent the recruitment of nucleic acids into endosomes, thereby suppressing immune activation.^90^ Consistent with this, loss of TIM3 through a germline mutation in *HAVCR2* results in the development of antibodies to double stranded DNA, which are usually a specific marker for SLE.^91^ Such germline mutations are present in around 60% of cases of subcutaneous panniculitis‐like T‐cell lymphoma, a condition associated with lupus‐like features in around 20% of cases. Reassuringly, phase II trials in metastatic lung cancer have demonstrated a side‐effect profile similar to that of pembrolizumab, although to date the nature of any irAEs, including whether any lupus‐like complications develop, has not been reported.^92^ Similarly germline loss of inhibitory TIM3 can result in a severe systemic inflammatory condition called haemophagocytic lymphohistiocytosis (HLH) that can complicate rheumatic diseases such as adult onset Still's disease, but this has not been reported with anti‐TIM3 therapy to date.

### 5.3 CD40/CD40L

The CD40/CD40L costimulatory pathway is an important checkpoint in both IMRDs and cancer. SNPs in CD40 are associated with the risk of rheumatoid arthritis, SLE, ankylosing spondylitis and Kawasaki disease across multiple GWAS studies.^63^ It is expressed by APCs and B cells (among other cell types) and, through interactions with the latter, is important for the generation and maturation of antibody responses through germinal centre formation, isotype switching, and somatic hypermutation. Inhibiting this pathway is an attractive target for RA and the more intractable Sjogren's syndrome, and there are ongoing clinical trials in both. In Sjogren's, treatment with anti‐CD40 therapies have shown initial efficacy with a significant reduction in the GC‐associated chemokine CXCL13.^93^

In contrast, CD40 targeting therapies are being employed in cancer because of its role in activation and maturation of DCs. Tumors with minimal T‐cell infiltration are generally resistant to checkpoint immunotherapy, including common tumors of the breast, ovary, and prostate. CD40 agonistic therapies appear to be able to overcome this resistance in murine models^94^ and in Phase I trials, neoadjuvant treatment with a CD40 agonist antibody promoted mature DCs and T‐cell infiltration.^95^ Surprisingly, given its central role in both cancer and autoimmunity, although data is limited there are no trial reports of malignancy or irAEs to date.^96^

## 6 PERSISTENCE OF RHEUMATIC IRAES

The anti‐tumor immune response induced by checkpoint inhibitors can induce a long‐lasting, durable remission and at higher rates than other systemic therapies.^97^ This anti‐tumor response can persist after withdrawal of treatment suggesting a permanent remodeling of the immune response.^98^, ^99^ Furthermore this response can be long‐lasting; there is evidence that IFNg‐producing tumor‐associated clones persist up to 9 years after cessation of treatment.^100^ In this situation a reinvigorated T‐cell response is capable of clearing the tumor and, having been primed, can provide ongoing surveillance to prevent recurrence. Unfortunately this desirable persistent loss of tolerance to the tumor is mirrored by persistent loss of tolerance to self in some irAEs. Mechanistically chronic irAEs can be subdivided into two groups. First, those that are the result of “burnout”, where cells expressing the targeted antigen are effectively ablated.^101^ Among rheumatic irAEs and IMRDs, respectively, exocrine gland dysfunction and Sjogren's are examples where irreversible loss or damage or gland function leads to symptoms such as xerostomia that do not respond to immunosuppression. Alternatively both can result in a state of smoldering inflammation where chronic, non‐resolving disease is the norm, such as arthritis. Rates of persistence vary but following treatment with anti‐PD1 around 43% of patients develop a chronic irAE (defined as lasting >12 weeks), with arthritis having one of the highest rates of persistence (49%).^102^ Although cessation of ICI treatment can lead to cessation of the irAE, once breach of tolerance has occurred it may persist; recurrence of the same toxicity upon rechallenge is common.^103^, ^104^, ^105^ The factors that influence this balance between a transient, self‐resolving immune response and sustained autoreactivity are pertinent to clinical scenarios such as vaccination and autoimmunity.

Prevention of chronicity of IMRDs is an area of huge clinical interest. Strong evidence suggests early and aggressive anti‐inflammatory treatment improves outcomes and improves the chances of entering remission.^106^ Reductions in inflammation can be induced pharmacologically or, in preclinical disease, through interventions such as smoking cessation and weight loss. It remains unclear whether systemic inflammation can influence prognosis with ICI treatment with mixed results, though in some cases suggesting that high basal levels of IL6 and TNF, and their reduction with treatment, correlate with partial or complete response.^107^ In agreement with this there is evidence that treatment of irAE‐arthritis with biologic therapies, particularly anti‐TNF therapy, shortens the time to cancer progression, presumably preventing development of robust and persistent memory.^108^

## 7 CONCLUDING REMARKS

We are at an early point in the characterization of rheumatic irAEs, to determine a nomenclature and classification analogous to IMRDs. There remain numerous unanswered questions about their causes, their heterogeneity, and how they relate to IMRDs. It is unclear the extent to which the efficacy of immunotherapy in cancer can be separated from these complications; however, and they are likely to become an increasingly prevalent clinical challenge. They highlight the centrality of immune checkpoints, conserved across species for millions of years of evolution,^109^ at the intersection of tolerance and reactivity across the most important challenges in human health. As they develop there will be more shared learning between rheumatic irAEs and IMRDs as they help to shed light on the fundamental mechanisms of tolerance.

## CONFLICT OF INTEREST STATEMENT

The authors have no conflict of interest to declare.

## Data Availability

No data available.
